# The Hospital Saturday Fund

**Published:** 1894-01-27

**Authors:** 


### THE HOSPITAL SATURDAY FUND.

A special meeting of the delegates of this fund was held at the offices in Farringdon Road, on Saturday evening, for the purpose of making the usual annual awards to the various hospitals, &c., of the metropolis. Mr. R. B. D. Acland (the chairman of the Council) presided. The Secretary (Mr. W. G. Bunn) reported that the total receipts for the past year (exclusive of a balance from 1892) had amounted to £19,301. The report of the Distribution Committee, which was adopted, recommended that of this sum £17,778 should be apportioned (as against £17,000 last year) as follows: To 29 general hospitals, £6,374 15s.; to 61 special hospitals, £6,375 11s.; to 34 dispensaries, £1,073 3s.; to 17 convalescent homes, £1,482 8s.; to 20 miscellaneous institutions (including distribution and surgical appliance committees, also institutions for the gratuitous nursing of the sick poor in their own homes), £2,193 12s., &c. The number of institutions to participate is 161, as compared with 155 the previous year.

The following is the list of some of the awards: General Hospitals.—London, £907 19s.; Guy's, £562 9s.; St. George's, £417 10s.; Middlesex, £407 12s.; St. Mary's, £391 12s.; Seaman's, £339 9s.; Westminster, £332 8s.; North London (University College), £337 11s.; King's College, £290 8s.; Charing Cross, £241 9s.; Royal Free, £246 7s.; West London, £204 4s.; German, £182 4s.; Temperance, £158 7s.; North-West London, £140 18s.; Deaconesses' Institution, £120 7s.; Great Northern Central, £154 12s.; Metropolitan Free, £130 7s.; Homœopathic, £130 7s.; West Ham, £121 17s.; Miller, £107 18s.; French, £98 2s.; Poplar, £97 1s.; Italian, £85 13s.; Queen's Jubilee, £58 17s.; St. John's (Twickenham), £44 7s.; Sidcup Cottage, £25; Wimbledon Cottage, £20; and Enfield Cottage Hospital, £20. Special Hospitals.—Cancer (Brompton), £139 18s.; Brompton Hospital for Consumption, £510 6s.; City of London, £368 4s.; Infirmary for Consumption, £133 7s.; North London, £175 15s.; Royal (City Road), £249 16s.; Royal National (Ventnor), £117 12s.; Alexandra, £146 6s.; Cheyne, £63 15s.; East London, £164 6s.; Hospital for Sick Children (Great Ormond Street), £216 13s.; North-Eastern, £91 18s.; Dental of London, £28 15s.; National Dental, £21 6s Epilepsy.—Hospital for Epilepsy and Diseases of the Nervous System, £57 14s.; National (Queen's Square), £229 11s. London Fever, £98 2s. Lock.—Lock Hospital, £122 17s. Lying-in.—British, £75 9s.; City of London, £92 7s.

